# OmpU as a biomarker for rapid discrimination between toxigenic and epidemic *Vibrio cholerae* O1/O139 and non-epidemic *Vibrio cholerae* in a modified MALDI-TOF MS assay

**DOI:** 10.1186/1471-2180-14-158

**Published:** 2014-06-18

**Authors:** Armand Paauw, Hein Trip, Marcin Niemcewicz, Ricela Sellek, Jonathan ME Heng, Roos H Mars-Groenendijk, Ad L de Jong, Joanna A Majchrzykiewicz-Koehorst, Jaran S Olsen, Evgeni Tsivtsivadze

**Affiliations:** 1 Department of CBRN Protection, TNO, P.O. Box 45, Rijswijk 2280 AA, The Netherlands; 2 Military Institute of Hygiene and Epidemiology, 24-100 Pulawy, Lubelska 2, Poland; 3 Instituto Tecnológico La Marañosa, Spanish Ministry of Defence, San Martín de la Vega, Madrid, Spain; 4 Radiological Environmental Recovery Program, Department of Environment, Centro de Investigaciones Energ eticas, Medioambientales y Tecnologicas (CIEMAT, Madrid, Spain; 5 Norwegian Defence Research Establishment, P. O. Box 25, Kjeller N-2027, Norway

## Abstract

**Background:**

Cholera is an acute diarrheal disease caused by *Vibrio cholerae*. Outbreaks are caused by a genetically homogenous group of strains from serogroup O1 or O139 that are able to produce the cholera toxin. Rapid detection and identification of these epidemic strains is essential for an effective response to cholera outbreaks.

**Results:**

The use of ferulic acid as a matrix in a new MALDI-TOF MS assay increased the measurable mass range of existing MALDI-TOF MS protocols for bacterial identification. The assay enabled rapid discrimination between epidemic *V. cholerae* O1/O139 strains and other less pathogenic *V. cholerae* strains. OmpU, an outer membrane protein whose amino acid sequence is highly conserved among epidemic strains of *V. cholerae,* appeared as a discriminatory marker in the novel MALDI-TOF MS assay.

**Conclusions:**

The extended mass range of MALDI-TOF MS measurements obtained by using ferulic acid improved the screening for biomarkers in complex protein mixtures. Differences in the mass of abundant homologous proteins due to variation in amino acid sequences can rapidly be examined in multiple samples. Here, a rapid MALDI-TOF MS assay was developed that could discriminate between epidemic O1/O139 strains and other less pathogenic *V. cholerae* strains based on differences in mass of the OmpU protein. It appeared that the amino acid sequence of OmpU from epidemic *V. cholerae* O1/O139 strains is unique and highly conserved.

## Background

Cholera is an acute diarrheal disease caused by *Vibrio cholerae* that can be lethal within hours if left untreated. In 2011, a total of 589,854 cases were registered from 58 countries, including 7,816 deaths [1]. The severity, duration, and frequency of cholera epidemics appear to be increasing [2], indicating that cholera is a severe public health problem. In addition, *V. cholerae* is considered a category B bioterrorism agent by the CDC [3]. Infection usually occurs by consumption of contaminated water, the natural habitat of *V. cholerae*, or contaminated food. Within the *V. cholerae* species, over 200 serogroups have been identified but only serogroup O1 and O139 strains that are able to produce cholera enterotoxin (CT) and toxin-coregulated pilus (TCP) can cause epidemics. The toxigenicity of a *V. cholerae* strain depends on its ability to produce the CT, encoded by the *ctxAB* genes, and TCP, encoded by the Vibrio pathogenicity island (VPI) [4]. However, these virulence factors are also described in non-O1/O139 *V. cholerae* isolates without causing an epidemic threat [5]. Next, occasionally, other strains of *V. cholerae* may cause diarrhea, but they do not have epidemic potential [6]. Rapid detection and identification of threatening microorganisms is essential for an effective response to an infectious disease outbreak. Therefore, rapid discrimination between epidemic *V. cholerae* O1/O139 strains and other *V. cholerae* strains is crucial. Matrix-assisted laser desorption/ionization time-of-flight mass spectrometry (MALDI-TOF MS) is increasingly used for quick identification of bacteria and possesses advantages over conventional techniques in that it is fast, accurate, cheap and suitable for high-throughput identification [7-10]. The discriminatory power of MALDI-TOF MS in analysis of whole bacterial cell lysates overlaid with α-cyano-4-hydroxycinnamic acid as a matrix is usually sufficient to identify bacteria to the species level but may also be used to differentiate between strains belonging to one species if adequate protein extraction procedures are performed [11-15]. The aim of this study was to develop a MALDI-TOF MS assay able to discriminate between toxigenic and epidemic *V. cholerae* O1/O139 strains and other mostly non-O1/O139 isolates. To extend the measurable range of the MALDI-TOF MS and thereby increase the discriminatory power of the MS spectra, ferulic acid was used as a matrix [16,17]. The outer membrane protein OmpU was identified as a suitable biomarker for discriminating between toxigenic and epidemic strains and non-epidemic strains.

## Methods

### Bacterial strains

In total, 48 clinical and environmental isolates of *V. cholerae* and *Vibrio mimicus* (Table 1) were obtained from Instituto Tecnológico La Marañosa, Spanish Ministry of Defence, San Martín de la Vega, Madrid, Spain, Norwegian Defence Research Establishment, Kjeller, Norway, and Military Institute of Hygiene and Epidemiology, Pulawy, Poland (Table 1) [18-20]. The human isolates were all collected as part of standard patient care. The isolates were collected from different areas of the world. Thirty-three, three, and twelve isolates were serotyped as O1, O139, and non-O1/O139 serogroups, respectively. From the 33 serogroup O1 isolates, 18 were clinical isolates, 10 were environmental isolates, and five isolates were from an unknown source. Two serogroup O139 isolates were clinical isolates and one was of unknown origin. From the isolates not belonging to serogroup O1 or O139, two isolates were of clinical origin and the 10 remaining isolates were of environmental origin.

**Table 1:** ***V. cholerae *isolates analyzed in this study**

				**Presence (1) or absence (0) of virulence genes**							**Allelic variants of targeted genes in MLST**^**a**^						
**Strain no.**	**Aliases**	**Serogroup**	**Serotype**	***ctxAB***	***tcpA-R1***	***tcpA-R2***	**Year**	**Host**	**Geographic origin**	**MLST genotype (GT)**	***cat***	***dnaE***	***gyrB***	***lap***	***recA***	**MSP value**^**b**^	**Reference**^**c**^
080025/EY	Vib12, F 751	O1	Ogawa	1	1	0	1990	Human	Spain	1	1	1	1	1	1	2.48	[18,19]
080025/EZ	Vib13, F 752	O1	Ogawa	1	1	0	1990	Human	Spain	1	1	1	1	1	1	2.17	[18,19]
080025/FA	Vib14, F 753	O1	Ogawa	1	1	0	1990	Human	Spain	1	1	1	1	1	1	2.46	[18,19]
080025/FB	Vib15, F 754	O1	Ogawa	1	1	0	1990	Human	Spain	1	1	1	1	1	1	2.30	[18,19]
080025/FC	Vib16, F 755	O1	Ogawa	1	1	0	1990	Human	Spain	1	1	1	1	1	1	2.36	[18,19]
080025/FD	Vib17, F 756	O1	Ogawa	1	1	0	1990	Water	Spain	1	1	1	1	1	1	2.19	[18,19]
080025/FE	Vib18, F 758	O1	Inaba	0	0	0	1991	Water	Spain	2	13	0	5	8	12	2.38	[18,19]
080025/FF	Vib19, F 759	O1	Inaba	0	0	0	1991	Water	Spain	2	12	0	5	8	12	2.22	[18,19]
080025/FG	Vib20, F 760	O1	Inaba	0	0	0	1991	Water	Spain	2	12	0	5	8	12	2.22	[18,19]
080025/FH	Vib21, F 761	O1	Inaba	0	0	0	1991	Prawn	Ecuador	2	12	0	3	9	12	2.21	[18,19]
080025/FI	Vib22, F 763	O1	Inaba	0	0	0	1991	Prawn	Ecuador	2	13	0	3	9	13	2.19	[18,19]
080025/FJ	Vib23, F 762	O1	Inaba	0	0	0	1991	Prawn	Ecuador	2	12	0	3	9	12	2.32	[18,19]
080025/FK	Vib24, F 764	O1	Inaba	0	0	0	1991	Prawn	Ecuador	2	12	0	3	9	12	2.30	[18,19]
080025/FL	Vib25, F 766	O1	Ogawa	0	0	0	1992	Water	Spain	3	9	8	11	7	8	2.37	[18,19]
080025/FM	Vib26, F 768	O1	Ogawa	1	1	0	1992	Human	Spain	1	1	1	1	1	1	2.15	[18,19]
080025/FN	Vib27, F 767	O1	Ogawa	1	1	0	1992	Human	Spain	1	1	1	1	1	1	2.47	[18,19]
080025/FO	Vib28, F 765	O1	Inaba	0	0	0	1991	Prawn	Ecuador	2	13	0	3	9	12	2.25	[18,19]
080025/FP	Vib29	O1	Ogawa	1	1	0	1993	Human	Spain	1	1	1	1	1	1	2.18	[18]
080025/FQ	Vib30	O1	Ogawa	1	1	0	1993	Human	Spain	1	1	1	1	1	1	2.40	[18]
080025/FS	Vib32	O1	Ogawa	0	0	0	1994	Human	Spain	3	9	0	11	7	8	2.17	[18]
080025/FT	Vib33	O1	Ogawa	1	1	0	1994	Human	Spain	1	1	1	1	1	1	2.22	[18]
080025/FU	Vib34	O1	Ogawa	1	1	0	1994	Human	Spain	1	1	1	1	1	1	2.37	[18]
080025/FV	Vib35	O1	Ogawa	1	1	0	1994	Human	Spain	1	1	1	1	1	1	2.50	[18]
080025/FW	Vib36	O1	Ogawa	1	1	0	1995	Human	Spain	1	1	1	1	1	1	2.37	[18]
080025/FX	Vib37	O1	Ogawa	1	1	0	1995	Human	Spain	1	1	1	1	1	1	2.48	[18]
080025/GD	Vib43	O1	Ogawa	1	1	0		unknown	unknown	1	2	1	1	1	2	2.37	[18]
080025/GE	Vib44	O1	Inaba	0	0	1		unknown	unknown	3	9	0	11	7	0	2.45	[18]
FFIVC057	2/23	O1	Ogawa	1	1	0	1994	Epidemic	Italy	1	1	1	1	1	1	2.50	[20]
FFIVC058	2/26	O1	Ogawa	1	1	0	1994	Epidemic	Italy	1	1	1	1	1	1	2.46	[20]
FFIVC065	2/70	O1	Ogawa	1	1	0	1994	Epidemic	Albania	1	1	1	1	1	1	2.51	[20]
FFIVC129	ATCC 33655	O1	Hikojima	1	0	1	1979	unknown	unknown	1	2	1	1	1	2	1.99	[20]
FFIVC016		O1	Ogawa	1	0	1		unknown	unknown	1	2	1	1	1	2	2.39	[20]
14/2002/S		O1	Unknown	1	1	0		unknown	unknown	1	1	1	1	0	1	2.42	[20]
FFIVC130	ATCC51394	O139		1	1	0	1995	Human	India	1	1	1	1	1	1	2.37	[20]
FFIVC131	CDC2412-93	O139		1	1	0	1995	Human	USA	1	1	1	1	1	1	2.43	[20]
FFIVC133		O139		1	1	0	2003	unknown	unknown	1	1	1	1	1	1	2.49	[20]
080025/FR	Vib31	O141		1	1	1	1993	Human	Spain	singleton	8	7	3	2	9	2.24	[18]
FFIVC050		non O1/O139		0	0	0		Mussels	Norway	singleton	8	9	9	11	5	2.28	[20]
FFIVC084		non O1/O139		0	0	0	2003	Mussels	Norway	singleton	4	2	4	3	3	2.45	[20]
FFIVC114		non O1/O139		0	0	0	2004	Water	Norway	4	6	1	6	6	6	2.29	[20]
FFIVC115		non O1/O139		0	0	0	2004	Water	Norway	4	6	1	6	6	6	2.39	[20]
FFIVC137		non O1/O139		0	0	0		Human	Norway	singleton	7	5	8	10	4	2.41	[20]
2/110/2006		non O1/O139		0	0	0	1998	Water	Poland	5	10	4	2	12	4	2.25	[18]
3/110/2006		non O1/O139		0	0	0	1998	Water	Poland	5	10	4	2	12	4	2.42	[18]
4/110/2006		non O1/O139		0	0	0	2004	Water	Poland	singleton	11	0	13	0	11	2.38	[18]
14/110/2006		non O1/O139		0	0	0	1998	Water	Poland	singleton	5	3	10	4	7	2.37	[18]
17/110/2006		non O1/O139		0	0	0	1998	Water	Poland	6	3	6	7	5	10	2.47	[18]
22/110/2006		non O1/O139		0	0	0	2004	Water	Poland	6	3	6	7	5	10	2.26	[18]
070256/J	*V. mimicus* ATCC 33655	-		1	0	0				10	14	10	12	1	14	1.71	[18]

^a^“0” means no PCR product was obtained.

^b^MSP value: highest logarithmic value of the four generated MS-spectra score value compared to Biotyper reference library.

^c^Reference(s), in which the isolate is described previously.

### Confirmation of strain identification

Identification of the isolates at species level was confirmed by MALDI-TOF MS using Biotyper 2.0 (Bruker Daltonics GmbH, Bremen, Germany) [11]. Serogroup and serotype were confirmed using the *Vibrio cholerae* E Agglutinating Sera kit containing specific antisera O1 polyvalent agglutination serum, Inaba agglutination serum, and Ogawa agglutination serum (Remel Europe Ltd. Darford, Kent, United Kingdom) according to the manufacturer’s guidelines.

### Genotyping of isolates with multilocus sequence typing (MLST) analysis

MLST analysis was performed according to Teh *et al. *[21]. Internal gene fragments of *dnaE*, *lap*, *recA*, *gyrB*, and *cat* were PCR amplified and sequenced. The *gmd* gene was not included in the analysis due to low discriminatory power [21]. Each sequence variant of a locus was assigned a distinct allele number. In the case that no PCR product could be obtained for a specific allele, the number zero was assigned. The allele profiles were entered into BioNumerics version 6.6 software (Applied-Maths, Belgium) as character values, and the genetic relationship between isolates was constructed using the categorical coefficient and the Minimum Spanning Tree algorithm. Isolates that differed at two or fewer loci were considered genetically closely related, while single locus variants (SLV) were defined as having at least three alleles that were different from all other tested isolates.

Isolates were screened for the presence of the virulence genes *ctxAB* and *tcpA* by PCR [21]. Template DNA was obtained from supernatants of cell suspensions lysed by heating for 10 min at 95°C. Amplification of DNA fragments from *dnaE*, *lap*, *recA*, *gyrB*, *cat*, *ompU*, *ctxAB*, and *tcpA* was performed with a HotStar Taq MasterMix kit (Qiagen, Westburg b.v., Leusden, The Netherlands). The primers used were previously described by Teh *et al*. [21]. The *ompU* genes from 9 isolates (including three epidemic strains (080025/EZ [O1 Ogawa], FFIVC130 [O139], and FFIVC129 [O1 Hikojima]), six environmental isolates (FFIVC114, 080025/FE, 080025/FI, 080025/FL, 17/110/2006, and 2/110/2006) were amplified using the primers ompU-fw (5′-ACCTATTTCGATTGACGTGGC-3′) and ompU-rv (5′-ACATCCACCAAGAAACGTTGC-3′), which anneal approximately 80 bp up- and downstream of the *ompU* open reading frames. The PCR products were bidirectionally sequenced. DNA sequencing was performed by BaseClear B.V. (Leiden, The Netherlands).

### Sample preparation for MALDI-TOF MS analysis

*V. cholerae* isolates were grown for 16 h at 35°C on blood agar plates. Sample preparation for MALDI-TOF MS analysis of whole cell lysates was performed as previously described [11]. Each isolate sample was spotted eight times on the MALDI target. Four spots were overlaid with 0.5 μl of 10 mg/ml α-cyano-4-hydroxycinnamic acid (HCCA, Bruker Daltonics) in an acetonitrile/water solution (1:1) with 2.5% trifluoroacetic acid (Fluka/Aldrich, Stenheim, Germany). Four spots were overlaid with 0.5 μl of a matrix solution containing 12.5 mg/ml ferulic acid (Sigma-Aldrich), 17% formic acid and 33% acetonitrile (LC-MS grade, Fluka/Aldrich, Stenheim, Germany), hereafter referred to as FA+ [16,17]. Spots were dried at room temperature.

### Mass spectra acquisition

The mass spectra were acquired automatically on a Bruker Autoflex III smartbeam instrument (Bruker Daltonics) in linear mode. Spots overlaid with HCCA matrix were analyzed using the following parameters: 50% laser intensity, positive polarity, 350 ns PIE delay, acceleration voltage of 20 kV (source 1) and 18.7 kV (source 2), lens voltage of 8 kV, linear detector voltage of 1.522 kV, and 500 Da detector gating. Composite mass spectra were generated from 10 different positions per spot using, in total, 2,000 laser shots at each spot generated by a 200-Hz smartbeam laser (355 nm). The mass spectra were recorded in a mass/charge (*m/z*) range of 2,000 – 20,000. The parameters used for analysis of the spots overlaid with the FA+ matrix were: 80% laser intensity, positive polarity, 350 ns PIE delay, acceleration voltage of 20 kV (source 1) and 18.7 kV (source 2), lens voltage of 2.8 kV, linear detector voltage of 1.522 kV, and 4000 Da detector gating. Composite mass spectra were generated from 10 different positions per spot using, in total, 2,000 laser shots at each spot generated by a 200-Hz smartbeam laser (355 nm). The mass spectra were recorded in a *m/z* range of 4,000 – 80,000. The instrument was externally calibrated with a bacterial test standard (BTS, Bruker Daltonics) when analyzing HCCA-overlaid spots or peptide calibration standard II (Bruker Daltonics) when analyzing spots overlaid with FA+. To evaluate the reproducibility of the newly developed method, the entire test was repeated on a separate day.

### Data analysis MS spectra

The MS spectra obtained from the spots overlaid with the HCCA matrix were analyzed using MALDI Biotyper 2.0 software and Bruker’s security relevant library (Bruker Daltonics). These libraries together contain 83 reference spectra (MSPs) from various *Vibrio* species, including three *V. cholerae* strains and one *V. mimicus* strain*.* For each measurement, a logarithmic score value was determined by calculating the proportion of matching peaks and peak intensities between the test spectrum and the reference spectra of the database [11,13]. Identification at species level was based on the highest of the four logarithmic values [11]. All MS spectra obtained from spots overlaid with the FA+ matrix were analyzed using Matlab software (version R2011b). The spectra were first converted into the MZXML format using the Bruker Daltonics supplied software (CompassXport) and subsequently converted to the Matlab binary format using mzxml read procedure. Further processing was performed using the Matlab Bioinformatics toolbox (Version 4.0) routines such as resampling (msresample - mass range 10,000 to 50,000 Da and resampling to 5,000 data points), baseline subtraction (msbackadj), alignment on a peak mass of 11974 (msalign), which was present in the MS spectra of all *V. cholerae* isolates, normalization (msnorm) and visualization of spectra in a heat map. Peaks were automatically selected using standard peak selection algorithm (mspeaks - HeightFilter = 2). The highest peak in the region of 32.5 – 37.5 kDa per isolate was automatically identified.

### Protein identification by SDS-PAGE coupled to LC-MS/MS

Viable cells of the *V. cholerae* isolates FFIVC129, FFIVC130, 080025/EZ, 080025/FC, 080025/FE, 080025/FI, FFIVC137 and 17/110/2006 were resuspended in 50 μl phosphate-buffered saline and mixed with 50 μl Laemmli 2x sample buffer (Bio-Rad). Samples were incubated at 100°C for 10 minutes and analyzed by standard SDS-PAGE using a 12% polyacrylamide gel and Coomassie Brilliant Blue staining [22]. The most prominent protein bands in the mass range of 34 to 38 kDa were excised from the gel and subjected to in-gel trypsin digestion. Gel pieces were washed with pure water, destained with three rounds of washing in a mixture of 70% 25 mM NH_4_HCO_3_/30% acetonitrile (ACN) and dehydrated by 10 minutes of incubation in 100% ACN. After removal of ACN, gel pieces were incubated in 100 mM NH_4_HCO_3_/10 mM dithiothreitol for 30 min at 56°C followed by addition of iodoacetamide to a final concentration of 55 mM and 30 min of incubation at room temperature. Gel pieces were washed in 25 mM NH_4_HCO_3_, dehydrated by incubation in 100% ACN, placed in 50 μl 100 mM NH_4_HCO_3_ containing 10 ng/ml trypsin (from bovine pancreas, Sigma-Aldrich) and incubated overnight at 37°C. The remaining liquid was transferred to a clean tube, and peptides were extracted from the gel pieces by two rounds of 5 minute incubation in 50 μl 60% ACN, 1% trifluoroacetic acid in an ultrasonic bath (37 kHz).The combined fractions were dried in a SpeedVac, and the pellets were resuspended in 30 μl H_2_O. The samples were analyzed by liquid chromatography-tandem mass spectrometry using an Ultimate 3000 RSLnano LC system (Thermo Scientific, Sunnyvale, CA) coupled to an HCTultra ion trap mass spectrometer (Bruker Daltonics). Samples were injected onto an Acclaim C_18_ PepMap100 trapping column (Thermo Scientific) and washed with 100% buffer A (3% ACN in 0.1% formic acid) at 5 μl /min for 6 min. Peptides were separated on an Acclaim C_18_ PepMap RSLC column at a constant flow rate of 300 nl/min. An elution gradient of 3 to 40% buffer B (95% ACN in 0.1% formic acid) was applied over 48 min followed by an increase to 65% B in 10 min. The nanoflow LC was coupled to the mass spectrometer using a nano-electrospray ionization source. Eluting peptides were analyzed using the data-dependent MS/MS mode over a 300–1500 *m/z* range. The five most abundant ions in an MS spectrum were selected for MS/MS analysis by collision-induced dissociation using helium as collision gas. Peak lists were generated using DataAnalysis 4.0 software (Bruker Daltonics) and exported as Mascot Generic files. These files were searched against the NCBI database with *V. cholerae* as taxonomy using the Mascot (version 2.2.1) search algorithm (Matrix Science, London, UK). Trypsin was selected as the enzyme for digestion and up to one missed cleavage site was allowed. Carbamidomethyl cysteine was selected as a fixed modification, and oxidation of methionine was selected as a variable modification.

## Results

### Strain identification

Forty-eight isolates acquired from different strain collections (Table 1) and previously identified as *V. cholerae* were analyzed using MALDI-TOF MS and Biotyper 2.0 software (Bruker Daltonics). All strains were identified as *V. cholerae* with matching scores of 1.99 to 2.51 following the highest matching score rule [11]. As a control, one *V. mimicus* isolate was analyzed, which resulted in a matching score value of 1.71, indicating a ‘probable genus identification’. In addition, serogroup and serotype designations were confirmed using specific antisera.

### MLST analysis

**Figure 1:**
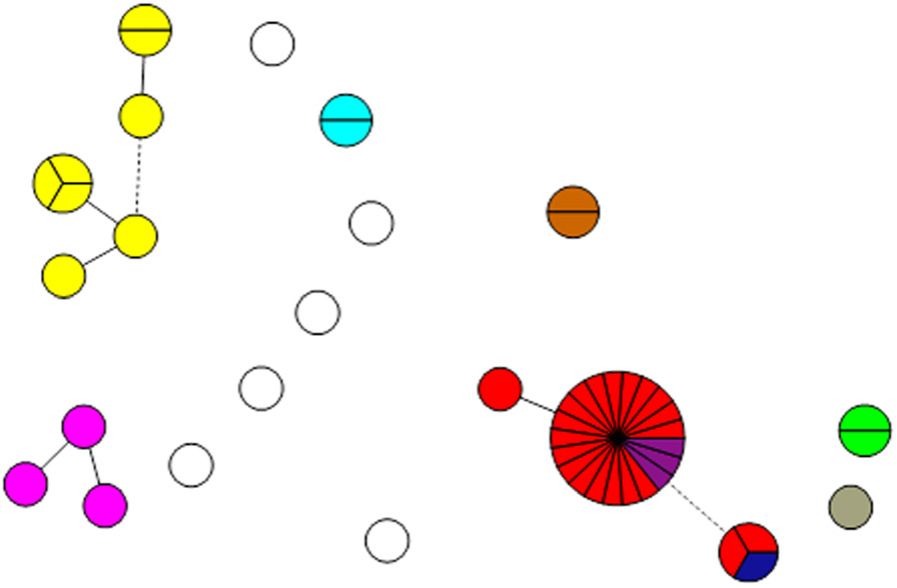
**Minimal Spanning Tree (MST) of *V. cholerae *isolates based on MLST data. Each circle corresponds to a sequence type.** The number of partitions in each circle corresponds to the number of isolates. Single locus variants are connected with a solid line; two single variants are connected with a dotted line. Red, serogroup O1 serotype Ogawa strains (GT1); purple, serogroup O139 (GT1); dark blue, serogroup O1 serotype Hikojima (GT1); yellow, serogroup O1 serotype Inaba (GT2); pink, serogroup O1 serotype Ogawa (2x) and Inaba (1x) (GT3). Green, brown and light blue, non-O1 or O139 serogroup strains (GT4, GT5, GT6). Gray, *V. mimicus*.

To determine the genetic relationship among the 48 *V. cholerae* isolates, a MLST analysis was performed. Accession numbers: *cat* KF421252 - KF421300, *dnaE* KF421301 - KF421338, *gyrB* KF421339 - KF421387, *lap* KF421388 - KF421434, and *recA* KF421435 - KF421482. The isolates were differentiated into six different genotypes (GT1-6) and six single locus variants (SLVs) (Table 1). The presence of the virulence genes *ctxAB* and *tcpA* was determined by PCR. All isolates of serogroups O1 or O139 that contained the *ctxAB* and *tcpA* were highly related (Figure 1). Within this group, all O1 isolates contained the type-specific antigen of the serotype Ogawa with the exception of one isolate that belonged to serotype Hikojima and one isolate of unknown serotype. *V. cholerae* O1 strains of serotype Hikojima are considered to be rare [23]. Isolates outside the GT1 group were determined to be negative for *ctxAB* with the exception of one SLV, an isolate of serogroup O141 that contained *ctxAB* and *tcpA*. Eight isolates of serogroup O1, serotype Inaba, isolated from water in Spain and from prawns in Ecuador were genetically closely related (GT2). Three other isolates of Spanish origin were genetically related (GT3). Furthermore, three pairs of closely related isolates were identified. Two pairs were isolated from the Bug river in Poland (GT5, GT6), while another pair was isolated in Norway from seawater near Oslo (GT4). Six SLVs from Spain, Norway and Poland were observed.

### MALDI-TOF MS analysis

**Figure 2:**
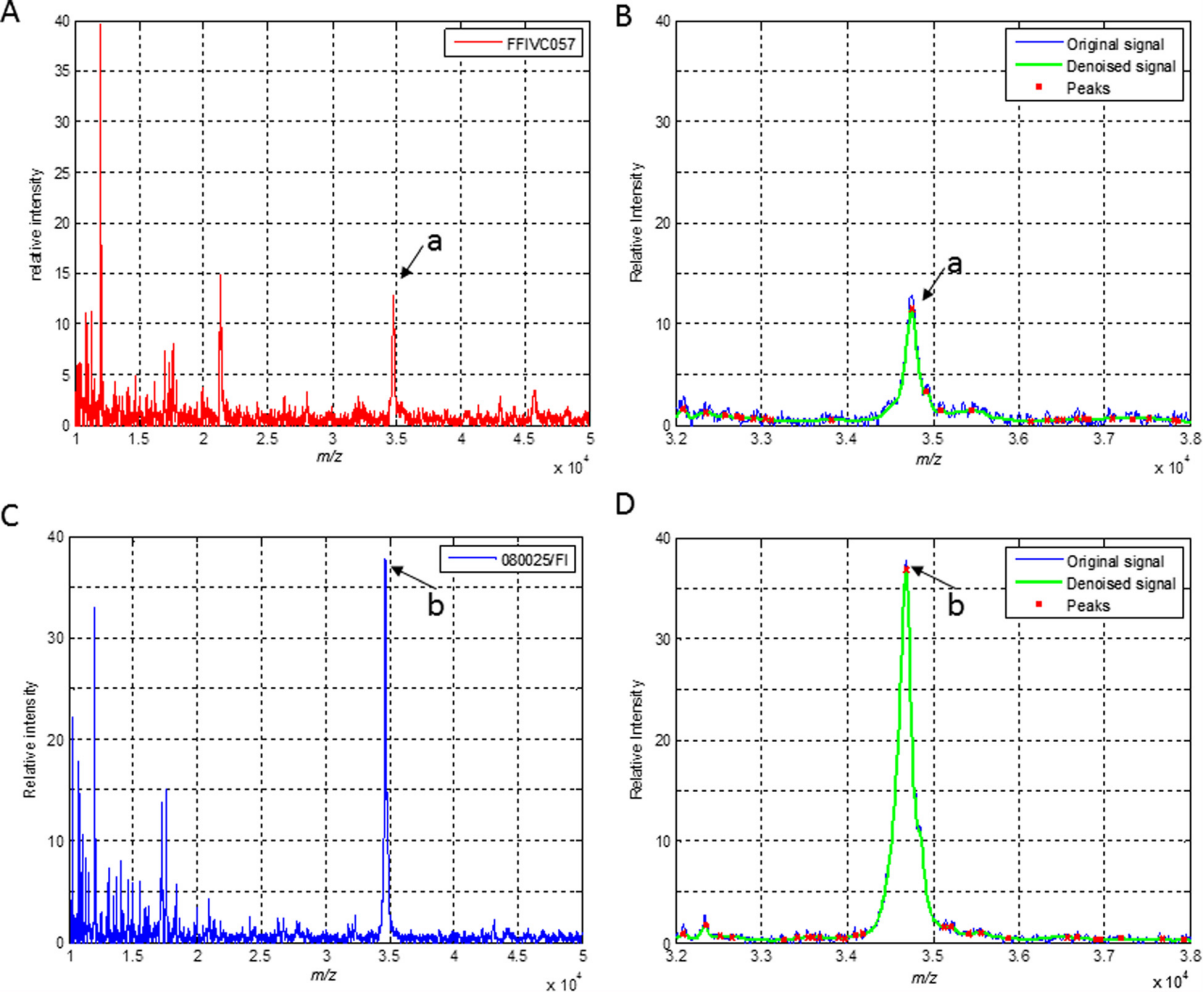
**MALDI-TOF MS analysis of whole cell lysates of *V. cholerae *isolates. A** and **B**, examples of normalized MS spectra of a toxigenic and epidemic serogroup O1 isolate (A) and a non-toxigenic isolate (B). **C** and **D**, close-ups of selected MS-peak of Figure **A** and **B**, respectively. a, m*/z* = 34,750 Da, b m*/z* = 34,690 Da.

**Figure 3:**
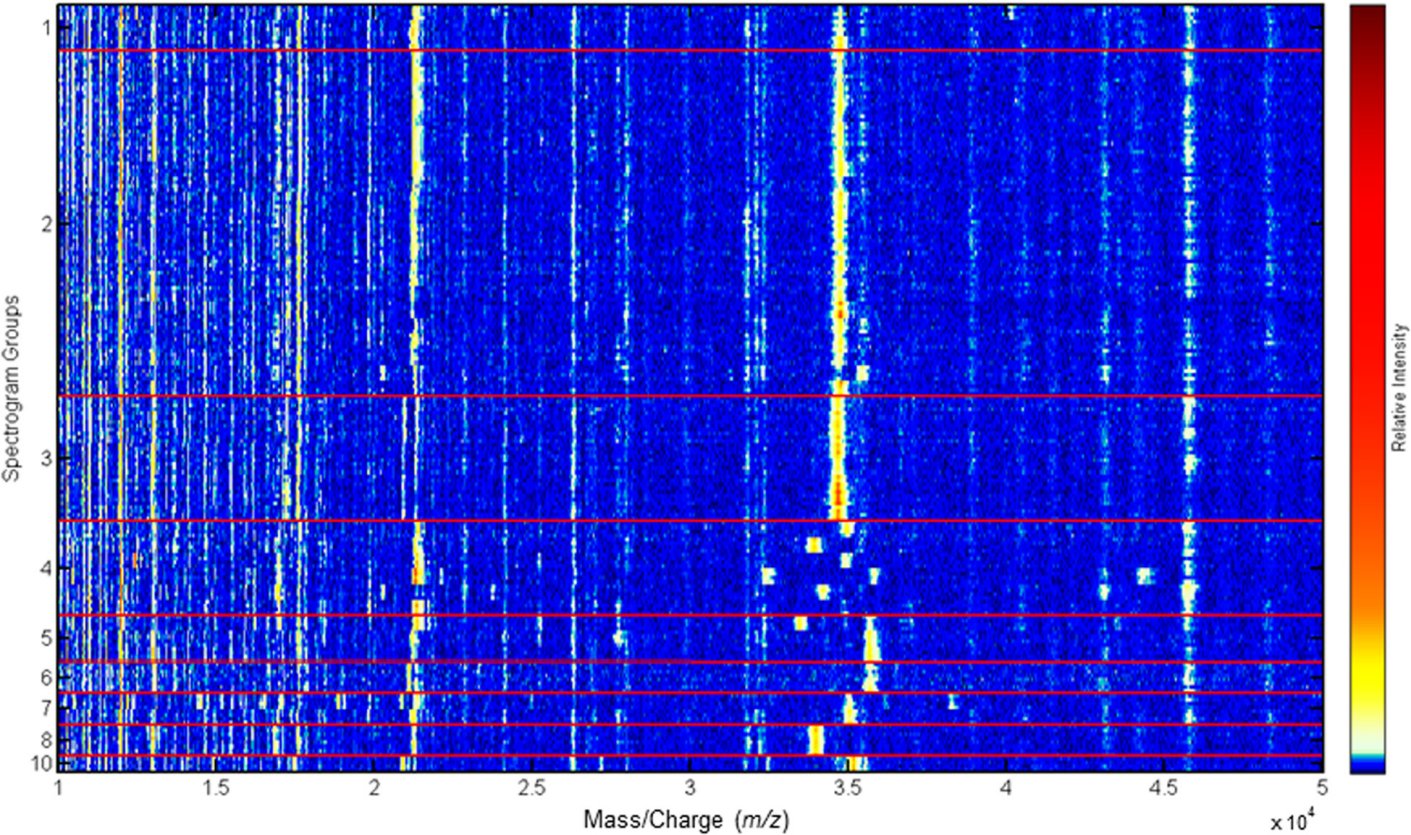
**Heat map analysis of MS spectra of 48 *V. cholerae *isolates and one *V. mimicus *strain.** Each isolate is represented by four spectra (horizontal lanes) obtained from four spots on the MALDI target. The color indicates the peak intensities according to the color scale (left bar). The spectra were divided into spectrogram groups (separated by red horizontal lines): 1, *V. cholerae* serogroup O139 (GT1); 2, *V. cholerae serogroup* O1 serotype Hikojima and Ogawa strains (GT1); 3, serogroup O1 serotype Inaba (GT2); 4, SLVs; 5, serogroup O1 serotype Ogawa (2x) and Inaba (1x) (GT3); 6 and 7, two pairs isolated from the Bug river in Poland (GT 4, GT5); 8, pair isolated in Norway (GT6); 10, *V. mimicus*.

**Figure 4:**
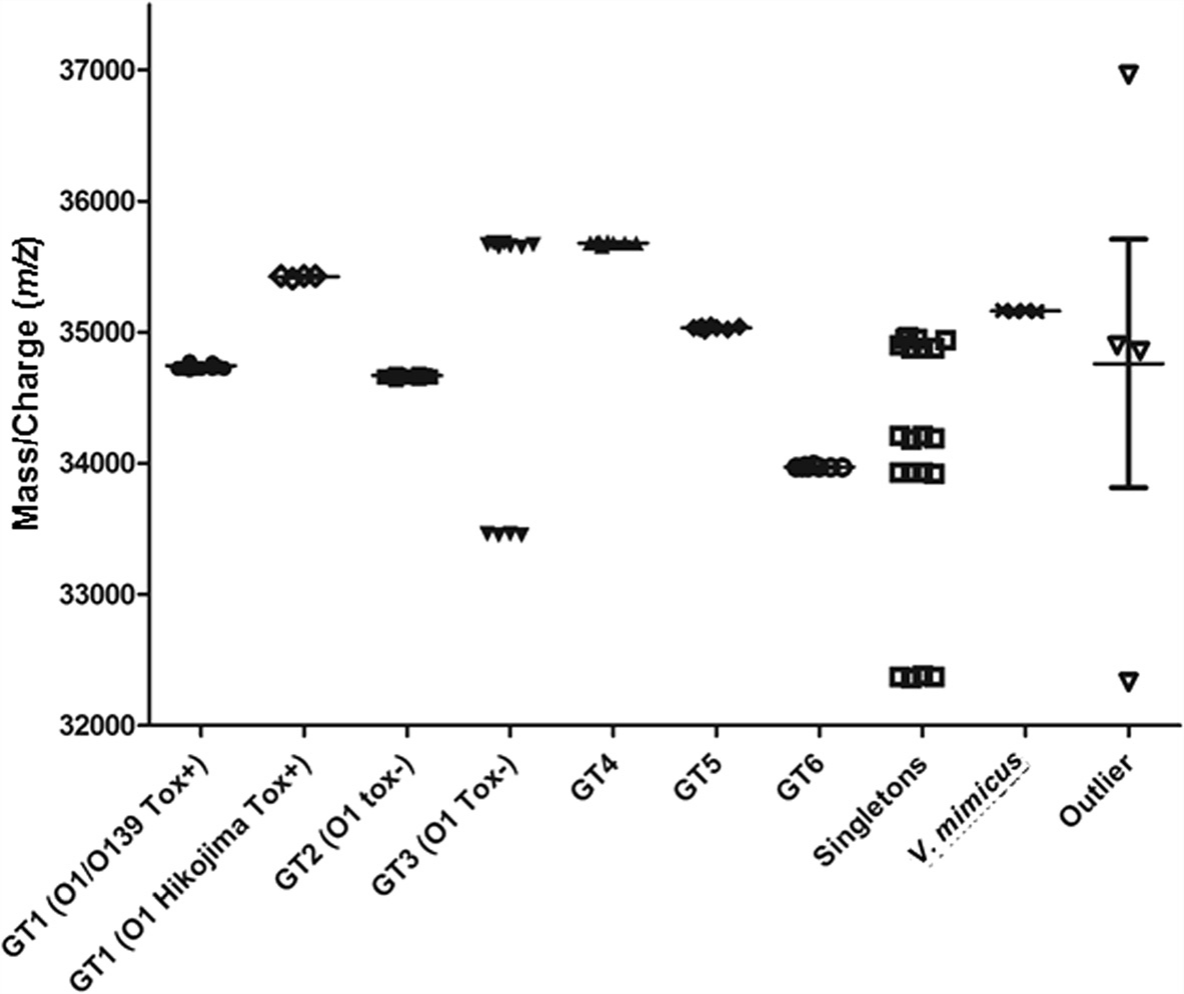
**Distribution of the highest-peak positions in the 32 to 38 kDa range grouped per genotype (GT).** Each isolate is represented by four peak positions. GT1 (O1/O139 Tox+) comprises 96 peak positions of 24 isolates; GT1 (O1 Hikojima Tox+) comprises 4 peak positions of 1 isolate; GT2 (O1, Tox-) 32 peak positions of 8 isolates; GT3 (O1 Tox-) shows 12 peak positions of 3 isolates with the same genotype but different serotypes. GT4, GT5 and GT6 each comprise 8 peak positions of 2 isolates; SLVs comprise 20 peak positions of 5 not related isolates; *V. mimicus* comprises 4 peak positions of one *V. mimicus* strain; Outlier comprises 4 peak positions of one outlier, in the second experiment for this isolate the maximal difference in peak positions was 52 Da.

To obtain spectra of a wider *m/z* range than acquired with HCCA as a matrix, whole cell extracts were analyzed with MALDI-TOF MS using FA+. Spectra were initially recorded in a mass-to-charge range of 4,000 to 80,000 (MZXML data available at http://www.learning-machines.com/). As no significant peaks were visible above an *m/z* value of 50,000, spectra were recorded up to *m/z* = 50,000 in following experiments (Figure 2). After the datasets were normalized, the baseline was subtracted, and data were aligned and normalized, a heat map was generated to visualize differences between the MS spectra (Figure 3). A simple algorithmic peak search procedure allowed us to identify a prevalent peak near an *m/z* value of 35,000 that appeared to be discriminatory among the different genotypes (Figure 3). In the spectra of all epidemic isolates of serogroups O1 and O139 (GT1), this peak corresponded to an average mass of 34,750 Da with a standard deviation of 22 Da except for the O1 serotype Hikojima strain (35,424 Da). In the spectra of the other isolates, the corresponding peak differed at least 70 Da from that of GT1 (Figures 3 and 4). The peaks that were closest to the peak mass of the GT1 spectra were those measured in the spectra of GT2, the non-epidemic *V. cholerae* O1 Inaba isolates related to a Spanish outbreak, which were 34,670 +/- 20 Da.

To test the reproducibility of the observed differences in the discriminatory peak masses, the experiment was repeated in a different manner in which isolates were randomly distributed into separate sets. The results for GT1 and GT2 are summarized in Table 2. The mean peak masses of the specific marker in the GT1 and GT2 isolates were 34,565 +/- 31 Da and 34,495 +/- 30 Da, corresponding to mean mass shifts of -185 and -175 Da, respectively, compared to the first experiment. This shows that in the *m/z* range near 35,000, the measured peak masses can deviate between separate experiments but that differences between different samples are relatively constant. By including an internal control of known mass, spectra can be calibrated. Reproducibility was further supported by the median of the GT1 and GT2 measurements, which were maximally 5 Da different from the mean, indicating a Gaussian distribution of the measurements.

**Table 2:** **MALDI-TOF MS data of selected biomarker peak (OmpU) of two genotype groups (GT1, toxigenic and epidemic *V. cholerae *O1/O139; GT2, non-toxigenic O1) obtained from two separate experiments**

			**m/z**			
	**GT 1**^**a**^			**GT 2**		
	**Exp1**	**Exp2**	**Δ Exp1,Exp2**	**Exp1**	**Exp2**	**ΔExp1,Exp2**
Mean	34750	34565	-185	34670	34495	-175
Median	34745	34565	-180	34670	34490	-180
Maximum Δ	25	30		15	30	
Minimum Δ	35	50		30	35	

^a^O1 Hikojima isolate not included.

### Identification of discriminatory peak as OmpU

**Figure 5:**
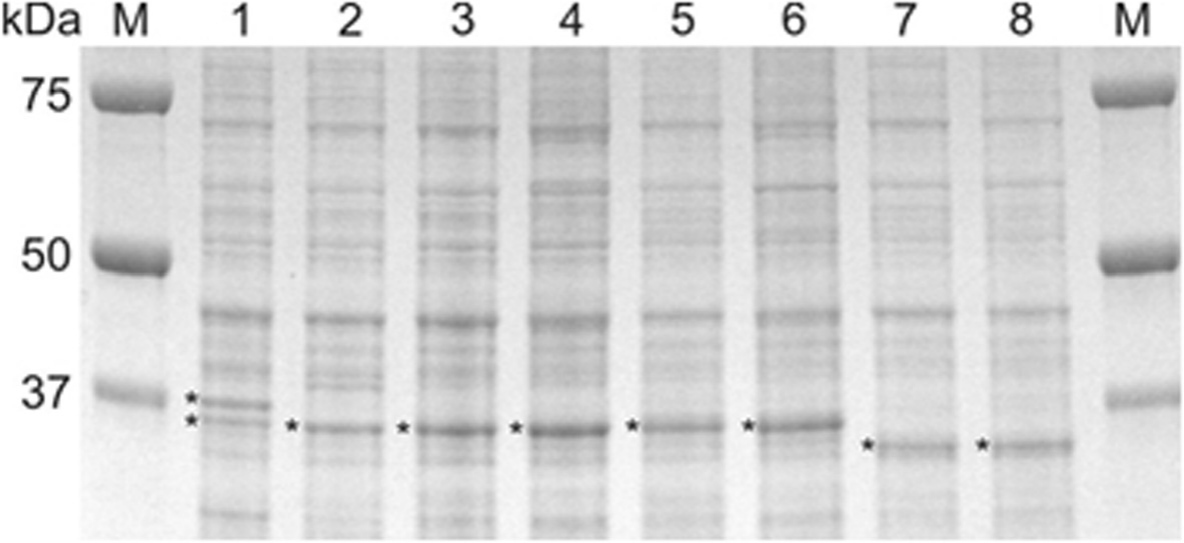
**SDS-PAGE analysis of whole cell fractions of eight *V. cholerae *isolates.** Lane 1, FFIVC129 ‘Hikojima’ isolate; 2, FFIVC130; 3, 080025/EZ; 4, 080025/FC; 5, 080025/FE; 6, 080025/FI; 7, FFIVC137; 8, 17/110/2006. Bands indicated with an asterisk were excised from the gel, in-gel digested with trypsin and analyzed by LC-MS/MS. All bands were identified as OmpU homologs except the upper band of strain FFIVC129 (*V. cholerae* O1 serotype Hikojima Tox + GT1), which was identified as OmpT.

One peak in the MS spectra representing the most abundant protein in a mass range of 30 to 40 kDa in *V. cholerae* cells grown overnight on rich medium agar plates was suggested to be a biomarker to differentiate between various *V. cholerae* strains. To identify this protein, whole cell lysates were analyzed by SDS-PAGE (Figure 5). Protein extracts from eight isolates of four different genotypes: GT1, 2, 6 and a SLV, were prepared from the same colony material that was used for MS analysis. One prominent band in the mass range of 32 – 37 kDa was present in the extracts of each of the isolates except for isolate FFIVC129, the ‘Hikojima strain’, which had two equally strong bands differing approximately 2 kDa in apparent mass. Differences in apparent masses in the SDS-PAGE analysis correlated with the differences of the peak masses in the MS spectra. The protein bands were excised, trypsin digested and analyzed by LC-MS/MS for identification. Of each band, the vast majority of peptides was identified as derived from OmpU homologs, except for the upper band of the Hikojima strain, which was identified as OmpT (Mascot 2.2.1 analysis). To confirm the correlation of the mass differences of the OmpU homologs with the peak mass differences, the *ompU* genes of 16 isolates were amplified and sequenced. (Accession numbers: KF434513 - KF434521 and KJ699296 - KJ699302). The theoretical masses of the mature OmpU homologs with omission of the signal peptide correlated with the observed peak masses of the MS spectra (less than 0.41% difference, Table 3) but not well enough to identify an epidemic isolate on basis of the measured peak mass alone. However, the theoretical mass differences between the isolates were consistent with the differences in the MS spectra within one experiment. The amino acid sequences of OmpU proteins from the epidemic *V. cholerae* O1 Ogawa and O139 isolates (080025/EZ and FFIVC130, respectively) were identical to the sequence of the OmpU protein from the epidemic type strain *V. cholerae* O1 El Tor Inaba N16961 (ATCC 39315) (Additional file 1: Figure S1). The OmpU protein from the *V. cholerae* O1 serotype Hikojima (isolate FFIVC129) differed at three positions (E290K, V324A, G325S) causing a mass difference of only one Dalton (OmpU N16961; 34,656 and OmpU FFIVC120; 34,657 Da). The OmpU proteins from the other tested strains deviated more from this sequence (Table 3). The OmpU proteins that were closest in mass were from the non-toxigenic outbreak isolates 080025/FE and 080025/FI (GT2), which differed at 9 positions, resulting in a 72 Da lower mass. The resolution of the MALDI-TOF MS spectra was sufficient to make this distinction (Table 3).

**Table 3:** **Theoretical and measured masses of OmpUs of 16** ***V. cholerae *isolates**

**Isolate**	**GT**		**Theoretical**			**Measured**	
					**1**^**st **^**exp**		**2**^**nd **^**exp**
		**mass**^**a**^	**Δ**^**b**^	**mass**^**c**^	**Δ ref**^**d**^	**mass**^**c**^	**Δ ref**^**d**^
080025/EZ	1	34656	0	34755	+ 6	34567	+ 12
FFIVC130	1	34656	0	34742	- 6	34543	- 12
FFIVC129	1	34657	+ 1	N.D.^e^		N.D.^e^	
FFIVC114	4	35595	+ 939	35683	+ 934	35506	- 951
080025/FE	2	34584	- 72	34672	- 77	34482	- 73
080025/FI	2	34584	- 72	34678	- 71	34508	- 47
080025/FL	3	35566	+ 910	35656	+ 907	35469	+ 914
17/110/2006	6	33871	- 785	33975	- 774	33733	- 822
2/110/2006	5	34961	+ 305	35031	+ 282	34875	+ 320
080025/FR	singleton	34870	+ 214	34951	+ 203	34784	+ 229
080025/GE	3	35566	+ 910	35670	+ 922	35501	+ 946
FFIVC050	singleton	33840	- 816	33924	- 824	33748	- 807
FFIVC084	singleton	34811	+ 155	34884	+ 136	34683	+ 128
FFIVC137	singleton	35709	+ 1053	35813	+ 1065	N.D.^f^	
4/110/2006	singleton	34122	- 534	34198	- 550	33977	- 578
14/110/2006	singleton	34826	+ 170	N.D.^f^		34716	+ 161

^a^Theoretical mass of mature OmpU in Da.

^b^Difference in mass with theoretical mass of OmpU of isolate 080025/EZ, in Da.

^c^Mean of peak masses obtained from 4 different MALDI spots.

^d^The average of OmpU peak masses of strain 080025/EZ and FFIVC130 was set as reference.

^e^N.D.: not determined, as OmpT instead of OmpU was assigned as the major peak in the 30000 – 40000 m/z range.

^f^N.D.: not determined because of failed measurement.

### OmpU is conserved among epidemic *V. cholerae* strains

Using BLASTp, the amino acid sequence of mature OmpU protein of *V. cholerae* N16961, which was used as a reference, was screened against the NCBI protein database (Table 4). At the time of preparation of this article, 181 *V. cholerae* OmpU homologs were present in the NCBI database. Ninety-six OmpUs were identical to the reference OmpU (from strain N16961) and these were all present in isolates of serogroup O1 or O139 that contain *ctxAB* and *tcpA*. One exception to this was a *V. cholerae* isolate of serotype O37 (strain V52), which was isolated during an outbreak in Sudan in 1968 (Table 4). This strain was shown to form a highly uniform clone together with *V. cholerae* O1 and O139 [24]. Two strains differed at one position from the reference OmpU. For one of these homologs, no strain information was provided. The OmpU of this isolate was 34 Da lower in mass compared to the reference OmpU. From the other isolate, CP1038(11), a *V. cholerae* O1 containing *ctxAB* and *tcpA* OmpU has a 58 Da higher mass than the reference OmpU from N16961 (Table 4). The OmpU proteins from two closely related *V. cholerae* strains of serogroup O1, the “Classical” biotype, RC27 (presence of *ctxAB* unknown, *tcpA* +*)* and O395 (*ctxAB* + and *tcpA*+*)*, were identical to that of the O1 Hikojima strain tested in this study (FFIVC129), having three amino acid mutations compared to N16961 OmpU, which results in a mass difference of 1 Da (Table 3). All other OmpU homologs retrieved in the BLASTp search contained ten or more mutations compared to the reference OmpU, resulting in a 58 Da lower mass in one case (strain BJG-01) or 70 Da or more difference in all other cases. The isolates harboring these OmpUs were all non-O1/O139 strains, with the exception of two O1 strains. However, no *ctxAB* or *tcpA* genes were found in the genome sequences of these strains, which strongly suggests that these are non-epidemic strains.

**Table 4:** **Results of BLASTp search using OmpU of *Vibrio cholerae *O1 El Tor N16961 (calculated molecular mass 34655.65 Da) as query sequence**

**Hit nr.**	**Mutations compared to OmpU N16961**	**Theoretical mass (Da)**	**Strain**	**Serogroup**	**Serotype**	**Biotype**	**Origin**	**Year of isolation**	**ctxAB**^**a**^	**tcpA**^**a**^	**Epidemic (E) or non-epidemic strain (N)**
1		34656	N16961	O1	Inaba	El tor	Bangladesh	1975	ctxAB+	tcpA+	E
1		34656	CP1032 (5)	O1	Ogawa	El tor	Mexico	1991	ctxAB+	tcpA+	E
1		34656	CP1044 (17)	O1^c^			Peru	1991	ctxAB+	tcpA+	E
1		34656	4260B	O139			Bangladesh	1993	ctxAB+	tcpA+	E
1		34656	CP1046 (19)	O1^c^			Peru	1995	ctxA+,ctxB^f^	tcpA+	E
1		34656	CP1047 (20)	O1^c^			Peru	1995	ctxAB+	tcpA+	E
1		34656	CP1033 (6)	O1^c^			Mexico	2000	ctxAB+	tcpA+	E
1		34656	CIRS101	O1	Inaba	El tor	Bangladesh	2002	ctxAB+	tcpA+	E
1		34656	CP1037 (10)	O1			Mexico	2003	ctxA+,ctxB-^f^	truncated	E
1		34656	CP1040 (13)	O1^c^			Zambia	2004	ctxAB+	tcpA+	E
1		34656	CP1041 (14)	O1	Ogawa	El tor	Zambia	2004	ctxAB+	tcpA+	E
1		34656	CP1030 (3)	O1^c^			Mexico	2008	ctxAB+	tcpA+	E
1		34656	**HC-06A1**^**e**^	O1	Ogawa	El tor	Haiti	2010	ctxAB+	tcpA+	E
1		34656	CP1042 (15)	O1	Ogawa	El tor	Thailand	2010	ctxAB+	tcpA+	E
1		34656	CP1048 (21)	O1	Ogawa	El tor	Bangladesh	2010	ctxAB+	tcpA+	E
1		34656	CP1050 (23)	O1^c^			Bangladesh	2010	ctxAB+	tcpA+	E
2^b^		34656	M66-2	O1	-	-	Indonesia	1937	ctxA+,ctxB-^f^	tcpA+	E
2		34656	MAK 757	O1	Ogawa	El Tor	Indonesia	1937	ctxAB+	tcpA+	E
2		34656	V52	O37			Sudan	1968	ctxAB+	tcpA+	E
2		34656	RC9	O1	Ogawa	El Tor	Kenya	1985	ctxAB+	tcpA+	E
2		34656	BX 330286	O1	Inaba	El Tor	Australia	1986	ctxAB+	tcpA+	E
2		34656	MO10	O139			India	1992	ctxAB+	tcpA+	E
2		34656	MJ-1236	O1	Inaba	El Tor	Bangladesh	1994	ctxAB+	tcpA+	E
2		34656	B33	O1	Ogawa	El Tor	Mozambique	2004	ctxAB+	tcpA+	E
3	F287I	34622	unknown	unknown		El tor			unknown	unknown	unknown
4	G325D	34714	CP1038 (11)	O1	Ogawa	El tor	Zimbabwe	2003	ctxAB+	tcpA+	E
5	E290K, V324A, 325S	34657	RC27	O1		Classical	Indonesia	1991	truncated	truncated	N
5	E290K, V324A, 325S	34657	O395	O1	Ogawa	Classical	India	1965	ctxAB+	truncated	N
7	10 mut	34598	BJG-01	non-O1^d^					ctxA+,ctxB-^f^	unknown	N
8	9 del , 13 mut	33840	HE-25	non-O1^d^			Haiti	2010	ctxAB -	tcpA -	N
9	9 del, 13 mut	33840	AM-19226	O39			Bangladesh	2001	ctxAB -	tcpA -	N
10	7 del, 18 mut	33911	RC385	O135			USA	1998	ctxAB -	tcpA -	N

^a^*ctxAB* and *tcpA* genes were identified by blastx search of whole genome sequences using *ctxAB* and *tcpA* of strain N16961 as query sequences.

^b^Hit nr. 2 represents OmpU identical to hit nr. 1 except for nine additional N-terminal residues resulting from a wrongly identified translation start.

^c^Presumed serotype O1 based on sequence similarity with O-antigen biosynthesis genes VC0241 to VC0244A from N16961.

^d^Presumed serotype non-O1/O139, based on lack of sequence similarity with O-antigen biosynthesis genes VC0241 to VC0244A from N16961 and O139. Accession: AB012956 bp 22084–24660 *wbfH*/*wbfI*/*wbfJ* ).

^e^This strain represents also 44 other *Vibrio cholerae* O1 El Tor Ogawa isolates from same outbreak with identical OmpU sequence and toxigenicity genes.

^f^No *ctxB* similar to *ctxB* of N16961 (locus_tag;VC1456). Presence of another variant of *ctxB* cannot be excluded.

In addition to the screening of OmpU homologs present in the NCBI protein database, 149 *ompU* sequences identified in completed whole genome sequences or whole genome shotgun (WGS) data of *V. cholerae* isolates available in the NCBI database were analyzed, and concomitantly, screened for the presence of the toxigenicity genes *ctxA* and *tcpA*. Based on sequence similarity with the O-antigen biosynthesis genes of O1 and O139 in N16961 and MO45, respectively, 108 strains were presumed O1 or O139. The amino acid sequence variation in OmpU in the 102 strains that also contained *ctxA* and *tcpA* was limited. In nine strains (including CP1038(11)) there was one amino acid difference compared to reference OmpU, resulting in 58 and 48 Da higher mass for eight strains and one strain, respectively. The variation in OmpU from six serogroup O1 isolates not harboring *ctxA* and *tcpA* differed 70 Da or more, similar to what was found with the BLASTp search. From the 41 analyzed non-O1/O139 strains the OmpU mass was in one case (strain BJG-01) 58 Da lower than that of the reference OmpU (see also BLASTp search) and in all other cases differed more than 70 Da.

It was shown that OmpU homologs differing 72 Da in theoretical mass (GT1 and GT2) could be well distinguished, as well as OmpU proteins from 080025/FL, 080025/GE (GT3) and FFIVC114 (GT4), which differed by only 29 Da in mass (GT3 (080025/FL, 080025/GE) and GT4 (FFIVC114)). Therefore, it can be assumed that OmpUs from epidemic strains (34,656 Da to 34,714 Da) can be distinguished from non-epidemic *V. cholerae* strains (less than 34,598 Da or more than 34,734 Da).

## Discussion

In this study, we demonstrate that the outer membrane protein OmpU from *V. cholerae* can be used as a biomarker of epidemic strains of *V. cholerae* in a new adapted MALDI-TOF MS assay. The use of ferulic acid as a matrix instead of α-cyano-4-hydroxycinnamic acid, commonly used in standardized MALDI-TOF assays for identification of bacteria, allowed for a larger measurable mass range (4 – 80 kDa), thereby including larger proteins such as OmpU (34 kDa) in the analysis. The resolution of the spectra was sufficient to discriminate between epidemic *V. cholerae* O1/O139 strains and other less pathogenic strains on the basis of mass differences in OmpU. OmpU appeared to be the dominant peak in an *m/z* range of 30,000 – 40,000 in the spectra of all 48 tested strains except for the spectrum representing the *V. cholerae* O1 strain of serotype Hikojima, where the most dominant peak was identified as OmpT. OmpU and OmpT are major outer membrane proteins of *V. cholerae *[25]. OmpU is expressed when cells are colonizing a human host, while OmpT is repressed at this time [26]. Reproducible differences between the OmpU peak masses of different MLST genotypes ranging from 32.4 to 35.7 kDa enabled discrimination of epidemic isolates from less or non-pathogenic isolates. Sequencing of the *ompU* genes in *V. cholerae* isolates representing different genotypes and a database analysis revealed that the amino acid sequence of OmpU from the epidemic *V. cholerae* O1/O139 and O37 strains is highly conserved, while OmpU homologs from other *V. cholerae* isolates varied from this sequence. These differences in amino acid sequence resulted in almost all cases in mass differences of more than 70 Da, which was sufficient to distinguish the “epidemic” OmpU proteins from OmpU proteins of other strains with the resolution of the method presented here. In general, differences in OmpU peak masses between strains were well reproducible in multiple experiments. However, small variations in the OmpU peak masses between separate experiments were observed, indicating that the method requires inclusion of a standard sample for calibration containing a characterized *V. cholerae* strain. Among the OmpU homologs of non-epidemic strains present in the NCBI database, one had a theoretical mass of 58 Da less than that of the “epidemic” OmpU protein, while in all other non-epidemic *V. cholerae* isolates the mass differed more than 70 Da. From the *in silico* analyzed 102 ‘epidemic’ isolates the theoretical mass of OmpU from eight, one and two isolates differed 58, 48 and 1 Da, respectively. Therefore, it can be assumed that epidemic strains (34,656 Da to 34,714 Da) can be distinguished from non-epidemic *V. cholerae* strains (less than 34,598 Da or more than 34,734 Da) based on OmpU using the described MALDI-TOF MS assay.

The *V. cholerae* strain of serotype Hikojima was shown to produce both OmpU and OmpT (Figure 5). However, in the obtained MS-spectra OmpU was not detected well and therefore its peak mass was not determined. More isolates of the Hikojima serotype, which is a rare serotype, need to be tested to determine whether this result is strain or serotype specific [23]. The theoretical mass of OmpU of the tested strain is only one Da less than that of the N16961 OmpU.

It should be noted that not all strains of serogroup O1 are toxigenic. Some strains are not able to produce the cholera toxin because these isolates lack the *ctxAB* and *tcpA* genes necessary for full virulence of *V. cholerae *[21,27]. Furthermore, the non-toxigenic O1 isolates in this study were also genetically distinct from the epidemic *V. cholerae* O1/O139 cluster (GT1), indicating that other unknown virulence factors could be present in the epidemic *V. cholerae* O1/O139 cluster that are absent in non-toxigenic *V. cholerae* O1 isolates. Previous studies have shown the presence of non-toxigenic *V. cholerae* O1 strains in the environment and in humans [6,18,21,27]. Serotyping is therefore not a reliable method for the identification of toxigenic and epidemic *V. cholerae* O1/O139 strains. Furthermore, *V. cholerae* non-O1/O139 isolates have been described that are able to produce the cholera toxin but are not considered epidemic because only strains of serogroup O1/O139 and O37 are able to cause large outbreaks [6,21,27]. Thus, the presence of the *ctxAB* and *tcpA* genes is not the only prerequisite for epidemic potential.

We have found that OmpU from epidemic *V. cholerae* has a unique and conserved amino acid sequence, which not only can be used in the presented MALDI-TOF MS assay, but also in a targeted PCR method. The difference in OmpU sequences between epidemic and non-epidemic isolates as well as the sequence variation among non-epidemic strains raises the question of whether this variation is due to genetic drift or specific adaptation to different niches. From a DNA alignment of a 5,000 bp region surrounding the *ompU* gene of seven epidemic O1 and five non-toxigenic strains (Additional file 2: Figure S2), it became clear that the *ompU* gene has undergone a higher mutation rate compared to the surrounding genes and intergenic regions. This suggests that OmpU has been subject to selective pressure, possibly as a result of adaptation to particular niches. A role for OmpU in host colonization has been proposed, potentially in enhancing attachment to epithelia in the gut or conferring resistance to bile, ionic detergents and organic acids [28-31]. Based on a three-dimensional model of *V. cholerae* OmpU, most of the variable regions are located in regions exposed to the outside of the cell (not shown), which supports a host-dependent variation hypothesis.

## Conclusions

Each year more than half a million people develop cholera. To reduce the burden of this devastating disease, new strategies must be developed. By minimizing the spread of the pathogen, the disease incidence can be reduced. To control a cholera outbreak, quick identification at the start of a potential outbreak and rapid discrimination between epidemic *V. cholerae* and other *V. cholerae* isolates could be helpful in introducing effective hygienic measurements [32,33]. To this point, discrimination between the toxigenic and epidemic *V. cholerae* strains and the non-pathogenic or less pathogenic strains has required multiple tests. The deviation in amino acid sequences of OmpU homologs of non-epidemic strains from those of the OmpU protein of strain N16961, which is conserved among almost all epidemic strains, makes OmpU an important biomarker to discriminate between epidemic *V. cholerae* O1/O139 and other *V. cholerae* isolates. The mass differences of OmpU proteins resulting from this sequence variation together with the high abundance of this protein in bacteria allows for the use of MALDI-TOF MS analysis as a rapid and discriminatory method for identifying epidemic strains of *V. cholerae*. Based on the described classification technique, one would maximally generate only one false negative classification when all characterized and sequenced *V. cholerae* isolates are screened with the developed MALDI-TOF MS assay.

## Competing interests

All authors declare that they have no competing interests.

## Authors’ contributions

Conceived and designed the experiments: AP HT, JAMK, ET. Performed the experiments: AP, HT, MN, RS, JMEH, RHMG, ALJ, JSO. Analyzed the data: AP, HT, ET. Contributed reagents/materials/analysis tools: AP, MN, RS, JSO, ET. Wrote the paper: AP, HT, MN, RK, JAMK, JSO, ET. Contributed to hypothesis generation and overall study design: AP, HT, JAMK, ET. All authors read and approved the final manuscript.

## Supplementary Material

Additional file 1: Figure S1 Alignment of OmpU sequences. The *ompU* genes from 16 isolates were sequenced. The translated OmpU amino acid sequences and the OmpU sequence of O1 El Tor strain N16961 were aligned using ClustalW software. Click here for file

Additional file 2: Figure S2 Alignment of 5 kbp DNA fragments of *ompU* loci from five non-toxigenic strains (1–6) and seven toxigenic O1 strains (7–13). Black vertical lines and regions indicate non-conserved bases. The upper green bar indicates conservation in the consensus. The diagram was made using Geneious software. *rrmJ*, 23S rRNA methyltransferase J; *greA*, transcription elongation factor GreA; *ompU*, outer membrane protein OmpU; *dacB*, D-alanyl-D-alanine carboxypeptidase/endopeptidase; *tyrS-2*, tyrosyl-tRNA synthetase. Click here for file
