# Supplementary figures and images for: OmpU as a biomarker for rapid discrimination between toxigenic and epidemic Vibrio cholerae O1/O139 and non-epidemic Vibrio cholerae in a modified MALDI-TOF MS assay
Source: BMC Microbiol. 2014 Jun 18;14:158. doi: 10.1186/1471-2180-14-158 (PMC4078931; doi:10.1186/1471-2180-14-158)

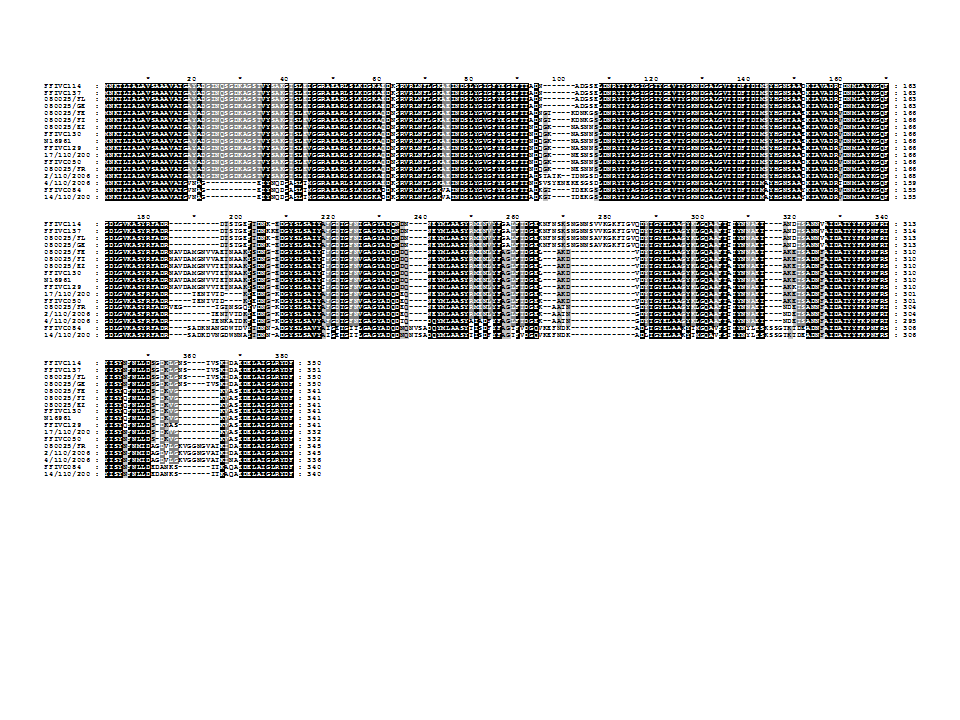

Supplement: Additional file 1: Figure S1 — Alignment of OmpU sequences. The ompU genes from 16 isolates were sequenced. The translated OmpU amino acid sequences and the OmpU sequence of O1 El Tor strain N16961 were aligned using ClustalW software. [file 1471-2180-14-158-S1.tiff]

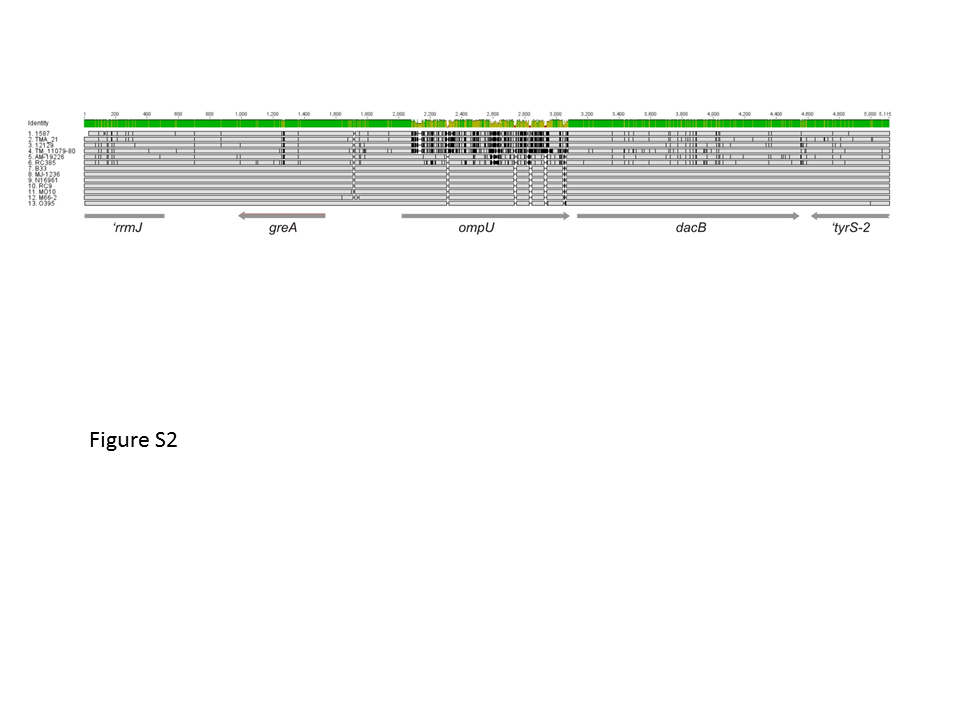

Supplement: Additional file 2: Figure S2 — Alignment of 5 kbp DNA fragments of ompU loci from five non-toxigenic strains (1–6) and seven toxigenic O1 strains (7–13). Black vertical lines and regions indicate non-conserved bases. The upper green bar indicates conservation in the consensus. The diagram was made using Geneious software. rrmJ, 23S rRNA methyltransferase J; greA, transcription elongation factor GreA; ompU, outer membrane protein OmpU; dacB, D-alanyl-D-alanine carboxypeptidase/endopeptidase; tyrS-2, tyrosyl-tRNA synthetase. [file 1471-2180-14-158-S2.tiff]
